# Health-related quality of life after deep vein thrombosis

**DOI:** 10.1186/s40064-016-2949-z

**Published:** 2016-08-08

**Authors:** Kristin Kornelia Utne, Mazdak Tavoly, Hilde Skuterud Wik, Lars Petter Jelsness-Jørgensen, René Holst, Per Morten Sandset, Waleed Ghanima

**Affiliations:** 1 Department of Medicine, Østfold Hospital Trust, Kalnes, Norway; 2 Institute of Clinical Medicine, University of Oslo, Oslo, Norway; 3 Department of Medicine, Sahlgrenska University Hospital, Gothenburg, Sweden; 4 Department of Haematology, Oslo University Hospital Rikshospitalet, Oslo, Norway; 5 Department of Health Science, Østfold University College, Fredrikstad, Norway; 6 Institute of Regional Health Research, University of Southern Denmark, Odense, Denmark; 7 Department of Haematology, Østfold Hospital Trust, Postbox 300, 1714 Grålum, Norway

**Keywords:** Controls, Deep vein thrombosis, EQ-5D-3L, HRQoL, Population norms, VEINES-QOL/Sym

## Abstract

**Background:**

Health-related quality of life (HRQoL) is known to be impaired in patients who develop post-thrombotic syndrome (PTS) following deep vein thrombosis (DVT). However, there is limited knowledge of the long-term HRQoL after DVT compared to controls without DVT. The objectives of this study were to evaluate long-term HRQoL following DVT and to compare that with age and sex matched control group and to population norms as well as to investigate possible predictors for reduced HRQoL.

**Methods:**

HRQoL was evaluated in 254 patients with confirmed DVT using the generic EQ-5D and the diseases specific VEINES-QOL/Sym questionnaire, whereas PTS was assessed by the Villalta scale. Patients were asked to give the EQ-5D questionnaire to two friends of same age- (±5 years) and sex (buddy controls).

**Results:**

Patients scored significantly lower on all dimensions of EQ-5D compared to controls. EQ-5D index value was lower in patients compared with buddy controls; mean 0.79 (SD 0.17; IQR 0.72–1.00) versus 0.9 (SD 0.12; IQR 0.80–1.00), p < 0.001. EQ-5D index value was also significantly lower than age- and sex-adjusted population norms (p < 0.001). PTS and obesity (BMI >30/m^2^) were significantly associated with impaired HRQoL assessed by EQ-5D index value (odds ratio [OR] 11.0: 95 % confidence interval [CI] 4.6–29.7; and 2.3: 95 % CI 1.1–4.8, respectively) and VEINES-QOL (OR 28.2: 95 % CI 10.6–75.0; and OR 4.1: 95 % CI 1.7–9.7, respectively).

**Conclusion:**

Long-term HRQoL was significantly impaired in DVT patients compared with buddy controls and population norms. PTS and obesity were independently associated with impaired HRQoL.

## Strengths and limitations of this study

Strengths

- Unselected DVT patients.
- Age- and sex-matched population norms and a control group were used for comparison with patients.
- Two validated measures for health-related quality of life (HRQoL) were used as the outcome, one for generic and one for disease specific HRQoL.

Limitations

- Patients were identified retrospectively.
- Incomplete response of buddy controls and coverage with the disease specific questionnaire.
- Patients were seen at a single time-point.

## Background

Deep vein thrombosis (DVT) is a common medical emergency estimated to affect about 460,000 people in the European Union countries each year (Cohen et al. 2007). Basic treatment of DVT comprises use of anticoagulants to block the activation of coagulation and to prevent thrombus extension (Kearon et al. 2012). Despite adequate anticoagulation, symptoms of DVT may take months to subside, and many patients develop long-term sequel. Generally, short- and long-term outcomes of DVT can be divided into those related to the treatment, such as bleeding, and those related to direct or indirect effects of the thrombus, such as pulmonary embolism, post-thrombotic syndrome (PTS) and recurrent venous thrombosis (Prandoni et al. 1996). PTS is a syndrome that affects up to 50 % of patients after DVT and is characterized by varying grades of pain, heaviness, swelling, and at worst, chronic ulceration of the leg (Ashrani and Heit 2009). PTS have been shown to negatively impact health-related quality of life (HRQoL) after a DVT (Kahn et al. 2014; Baldwin et al. 2013).

HRQoL assessed by patient-reported outcome measures (PROMs), typically obtained by questionnaires, provide essential information regarding the impact of diseases and treatments on patients’ physical-, psychological-, and social-functioning (Paller and Smith 2014). Comprehensive assessment of HRQoL should include the use of both generic and disease-specific measures (Lamping et al. 2003).

Although many studies have been published on HRQoL after DVT, most of these studies have focused on the impact of PTS on HRQoL (Ghanima et al. 2011; Kahn et al.  2005; Catarinella et al. 2014), whereas little is known on the long-term impact of DVT on HRQoL as compared to the general population. The primary aim of this study was thus to evaluate long-term HRQoL following DVT compared to population norms and age- and sex-matched controls as well as to investigate possible predictors of impaired HRQoL in these patients.

## Materials and methods

### Patient population

**Fig. 1:**
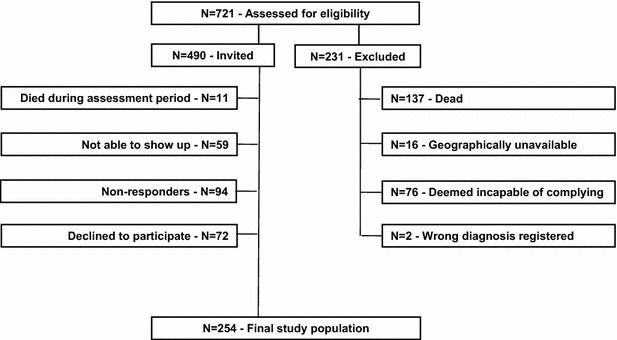
Flow-chart of patient sample

Patients that were diagnosed and treated for DVT at the Østfold Hospital, Norway, between 2004 and 2014 were identified from Østfold Hospitals Thrombosis Registry and by searching hospital databases for ICD-10 codes of DVT (I80.1, I80.2 and I80.3). We identified 721 patients with an objectively confirmed diagnosis of DVT (by compression ultrasonography), of whom 137 were deceased and 94 patients were excluded prior to invitation for reasons presented in Fig. 1. In total 490 patients were invited to participate. The inclusion period ran from 2012 to 2014. As shown in Fig. 1, 72 invited patients declined to participate, 59 were not able to show up for examination, 94 did not respond to the invitation, and 11 died during assessment period. Thus, the final study population comprised 254 patients.

### Methods

#### Clinical and epidemiological data

This population-based, cross-sectional study was designed to evaluate HRQoL among patients who had sustained one or more DVTs during the previous 1–10 years. Patients met at the hospital for a clinical evaluation including assessment of PTS by the Villalta scale. Information on localization of DVT, recurrent DVT(s), comorbidities, body mass index (BMI), sociodemographic data, presence of provoking factor, use of elastic compression stockings (ECS), and type and duration of anticoagulant treatment were acquired from the patients during the interviews or from the medical records.

#### Assessment of PTS

PTS was diagnosed in the index leg by the Villalta score (Kahn et al. 2009). This score includes the five patient-rated symptoms: pain, cramps, heaviness, paraesthesia, and pruritus; and the six clinician-rated signs: edema, skin induration, hyperpigmentation, pain during calf compression, venous ectasia, and redness. Each sign or symptom is rated as 0 (none), 1 (mild), 2 (moderate) or 3 (severe), and summed to produce a total score. Severity of PTS is divided into the following 4 groups depending on score; 0–4 points indicates no PTS, 5–9 points indicates mild-, 10–14 points indicates moderate-, and >14 points indicates severe PTS.

#### Health-related quality of life (HRQoL)

HRQoL was assessed using two questionnaires; EQ-5D and VEINES-QOL/Sym. Patients were sent the questionnaires by mail and were asked to bring back completed forms to the scheduled visit.

EQ-5D is a standardized and widely used instrument to assess generic HRQoL (EuroQol 1990; Brooks 1996). It is a descriptive system of HRQoL states consisting of five dimensions (mobility, self-care, usual activities, pain/discomfort, anxiety/depression), each of which can be scored according to three levels of severity (no problems/moderate problems/extreme problems). EQ-5D health states, defined by the EQ-5D descriptive system, may be summarized as a weighted mean of the five dimensions (Sorensen et al. 2009; Kim et al. 2008). The EQ-5D index value scores assigns each health state a value ranging from −0.59 to 1.00 where 1.00 indicates perfect health, 0.00 indicates death and negative scores indicates health worse than death. The questionnaire contains in addition the EQ Visual Analogue Scale (EQ VAS), which is scored from 0 to 100—from the “worst imaginable health state” to the “best imaginable health state”.

VEINES-QOL/Sym is a disease specific HRQoL instrument for use in chronic venous diseases of the leg (CVDL). The questionnaire consists of 26 items. It includes questions about symptoms due to CVDL (ten items), limitations in daily activities due to CVDL (nine items) and psychological impact (five items), as well as questions asking about the amount of change in the respondent’s leg problem over a 1-year period (one item) and the time of day that the leg problem is most intense (one item). Responses are rated on 2-point to 7-point descriptive scales, and two summary scores are computed, VEINES-QOL and VEINES-Sym. These scores were computed using standard scoring algorithms obtained from the authors (Lamping et al. 2003). 195 (77 %) patients returned a complete VEINES questionnaire.

#### Population norms and controls

HRQoL among the DVT patients was compared to the general Danish population by comparing the EQ-5D index value in the study sample with age- and sex-adjusted Danish population norms. Weighted time-trade-off (TTO) values were calculated by multiplying the number of patients per defined age group with the age-appropriate EQ-5D index value, established by Sorensen et al. (Sorensen et al. 2009) in 2009. Mean value in each gender group was then calculated (Table 3). Danish population norms were chosen due to lack of Norwegian population norms for EQ-5D.

Using the population norm data as reference, we could not ascertain the absence of patients with a history of DVT in this control group. In order to do so, we included a second control group that was acquired by asking our study subjects to hand over an EQ-5D form to two friends or relatives without a history of venous thrombosis with the same age- (±5 years) and sex. These buddy controls were asked to answer the questionnaire, however, only 122 controls responded and returned HRQoL questionnaires.

HRQoL assessed by VEINES-QOL/Sym was not compared between patients and buddy-controls, as VEINES-QOL/Sym is disease specific questionnaire designed for patients with DVT.

#### Assessment of clinical significance

For evaluation of whether a statistical difference between patients and control groups actually represented a clinically significant difference we used a minimally important difference (MID) in EQ-5D index value of 0.074, based on recommendations in the literature (Walters and Brazier 2005). For EQ VAS the MID is not known and was defined as half the SD among the controls, based on recommendations in the literature (Pickard et al. 2007).

#### Predictors of HRQoL

We tested for the following possible predictors of reduced HRQoL based on clinical experience and information available in the literature (Kahn et al. 2004, 2007, 2008): age, sex, employment status, comorbidities (active or previous cancer, chronic obstructive pulmonary disease (COPD), heart failure, and), obesity, smoking, provoked or unprovoked DVT, recurrent DVT, localization of DVT, ongoing anticoagulation, obesity, PTS, and use of ECS.

Index DVT was defined as the first thrombosis recorded in the hospital thrombosis registry. Proximal thrombosis was defined as a DVT in the popliteal vein or proximal, while DVT below the popliteal vein was considered distal. DVT was considered provoked if thrombosis was triggered by one of the following risk factors: orthopedic surgery, other extensive surgery, trauma or hospitalization with immobilization >3 days ≤12 weeks prior to index event, pregnancy or birth ≤12 weeks prior to index event, or long haul flight for over 4 h ≤12 weeks prior to index event. (Hansson et al. 2000). Recurrent DVT was registered when patient had sustained more than one DVT, before or after index event. Employment was categorized into either employed (all patients working or studying) or unemployed (all patients not attending any form of work).

#### Statistical analysis

Categorical variables were expressed as frequencies and proportions, while continuous variables were expressed by as mean and standard deviation (SD) when normally distributed and by median and interquartile range (IQR) when distribution was skewed. When comparing continuous variables deviating from the normal distribution between groups, non-parametric test (Mann–Whitney) was used. When comparing the EQ-5D index values between patients and controls, the results were presented as mean and standard deviation (SD), in addition to IQR, although the data deviate from normal distribution, since this is considered the standard method for reporting EQ-5D (Enden et al. 2007; Kwon and Kim 2016). All scores were consequently dichotomized into impaired/not impaired HRQoL. Impaired HRQoL was defined as scores <the 25th percentile among patients.

In the EQ-5D dimensions, the outcome “extreme problems” occurred very rarely, and this level was merged with the level “some problems” for the statistical analyses. Chi square test was used for comparison of the EQ-5D dimensions between patients and buddy-controls.

We performed univariate analysis using simple logistic regression to find possible predictors for impaired HRQoL. All variables, which were significantly associated with impaired HRQoL, were included in the multivariate models and backward variable selection was used. Wald test was used for assessment of significance in the logistic regression analyses. The issue of multiple testing was handled by a Bonferroni correction. A significance level of 2.5 % was therefore used for all tests, giving a joint significance level of 5 % (alpha 2.5 %).

Statistical analyses were performed using the Statistical Package for Social Science version 21.0 (SPSS Inc., Chicago, IL, USA).

## Results

The patients’ characteristics are summarized in Table 1. Mean age of the 254 patients at inclusion was 60 (SD 13) years and 167 (66 %) were males. Mean observation time from diagnosis of DVT (index event) to study inclusion was 5 (SD 2) years. Seventy-nine patients (31 %) had experienced more than one DVT; 36 (14 %) patients had DVT before and 43 (17 %) after the index DVT (Table 1). Forty-six patients (19 %) had ipsi-lateral recurrent DVT, before or after index DVT. One hundred and twenty-one patients (48 %) were diagnosed with PTS; but only 12 (5 %) with severe affliction (Table 1).

**Table 1:** Socio-demographic and clinical characteristics of the study sample at the time of inclusion in the study

Variable	Study sample (n = 254)	
	N (%)	
*Demographics*		
Male	167 (66)	
Age, mean (SD)	60 (13)	
Unemployed	135 (53)	
Years since diagnosis, mean (SD)	5 (2)	
Time on anticoagulation (months), median (IQR)	6 (3–34)	
Index DVT in left leg	132 (52)	
Proximal DVT^a^	161 (64)	
Recurrent DVT	79 (31)	
Still on anticoagulation therapy	74 (29)	
Any use of ECS after DVT	233 (93)	
PTS^b^	121 (48)	
*Comorbidities*		
Current smoker or previous smoker	131 (53)	
Current or previous cancer	20 (8)	
Heart failure	12 (5)	
COPD	13 (5)	
Obesity	76 (30)	
*Provoking factors*		
Orthopedic surgery	25 (10)	
Other extensive surgery	8 (3)	
Trauma which caused immobilization	46 (18)	
Pregnancy or birth	12 (5)	
Hospitalization with immobilization	3 (1)	
Long haul flight	25 (10)	

*COPD* chronic obstructive pulmonary disease, *ECS* elastic compression stockings, *PTS* post thrombotic syndrome, *Obesity* body mass index >30, *SD* standard deviation

^a^The exact location of DVT was missing in 6 patients as these patients were diagnosed at another institution and were referred to Ostfold hospital for follow up

^b^72 patients (28 %) were diagnosed mild-, 37 (15 %) with moderate-, and 12 (5 %) with severe PTS

Mean age of the patients (n = 100) having a buddy control was 63 (SD 12) years at inclusion and 68 (68 %) were male. Mean observation time from DVT to study inclusion in this group was 4 (SD 2, range 1–10) years; 22 (22 %) had ipsi-lateral recurrent DVT.

The mean age of patients who completed the VEINES questionnaire (n = 195) was 61 (SD 13) at inclusion and 128 (66 %) were males. Mean observation time from diagnosis of DVT to study inclusion was 5 (SD 2, range 1–9); 35 (18 %) had ipsi-lateral recurrent DVT.

### HRQoL

Patients scored significantly lower than controls on all EQ-5D dimensions (Table 2). More than 60 % of patients complained about pain/discomfort, which was the most prevalent complaint as compared to 31 % of the buddy controls. Anxiety and depression was present in 29 % of patients as compared to 12 % in the buddy control group. Mean EQ-5D index value (0.79; SD 0.20; IQR 0.72–1.00) in patients was significantly lower than in buddy controls (0.91; SD 0.12; IQR 0.80–1.00), (p < 0.001). The difference of 0.12 was larger than 0.074, which was larger than the defined MID.

**Table 2:** EQ-5D-3L among patients and controls

	Patients (n = 254)	Controls (n = 122)	P^a^ (patients versus controls)
	Mean (SD)	Mean (SD)	
EQ-5D index value	0.79 (0.20)	0.91 (0.12)	<0.001
EQ VAS	72 (19)	82 (15)	<0.001

EQ-5D dimension	Patients with problems^b^	Controls with problems^b^	P^c^
	n (%)	n (%)	
Mobility	79 (31)	13 (11)	<0.001
Self-care	17 (7)	0 (0)	0.003
Usual activities	68 (27)	8 (7)	<0.001
Pain/discomfort	157 (62)	38 (31)	<0.001
Anxiety/depression	74 (29)	15 (12)	<0.001

*PTS* post-thrombotic syndrome, *SD* standard deviation

^a^Man Whitney U test

^b^In the EQ-5D questionnaire, the outcome ‘extreme problems’ occurred very rarely, and this level was merged with the level ‘some problems’ for the statistical analyses

^c^Chi square test for independence

The mean score for EQ VAS (72; SD 19) was significantly lower in patients compared to buddy controls (82; SD 15), (p < 0.001) (Table 2). The difference of 10 was larger than 0.5 SD in patient group, which was considered as MID.

The mean EQ-5D index values for females (0.76; SD 0.19; IQR 0.70–0.82) and males (0.80; SD 0.21; IQR 0.76–1.00) were significantly lower than the corresponding age-adjusted Danish population norms for females (0.84) and males (0.88) (p < 0.001 for both comparisons). For both genders the difference was 0.8 which was larger than the defined MID (Table 3).

**Table 3:** Weighted time to trade of (TTO) values for EQ-5D index value

Age group	<29	30–39	40–49	50–59	60–69	70–79	>80
*Male n* *=* *167*							
No of patients	1	2	18	52	59	21	14
Index value in Danish population	0.94	0.93	0.91	0.89	0.88	0.85	0.80
*Female n* *=* *87*							
No of patients	6	8	8	15	25	15	10
Index value in Danish population	0.92	0.90	0.88	0.86	0.84	0.82	0.72

Weighted TTO values were calculated by number of patients per age group multiplied by age appropriate EQ-5D index value, mean value in each gender group was calculated. TTO values to compare mean study sample values with: male 0.88, female 0.84

The mean score of VEINES-QOL was 47.8 (SD 10.5). The largest difference in VEINES scores was found when comparing patients with and without PTS. Mean score of patients with PTS was 40.6 (SD 10.29) compared to 54.2 (SD 5.5) in PTS free patients, p < 0.001. Obese patients had a mean score of 41.9 (SD 10.9) compared to 50.2 (SD 9.4), p < 0.001. Females scored significantly lower than males [45.2 (SD 11.4) and 49.3 (SD 9.8), p = 0.01]. Distal versus proximal location of DVT did not significantly influence VEINES-QOL, p = 0.5; neither did recurrent versus first time DVT, p = 0.2. Results for VEINES Sym were approximately the same as for VEINES-QOL (not shown).

### Predictors of impaired HRQoL

The cut-off for impaired HRQoL was 0.72 and 60 for EQ-5D index value and EQ-VAS, respectively. Obesity, PTS, and unemployment, were found to be associated with impaired EQ-5D index and EQ VAS scores in simple logistic regression analyses (Table 4).

**Table 4:** Crude- and adjusted odds ratios (OR) for impaired HRQoL assessed by EQ-5D index value and EQ-VAS

Variable	EQ-5D index value					EQ-VAS				
	N = 254	Crude OR (95 % CI)	p value^#^	Adjusted OR^a^ (95 % CI)	p value^#^ adjusted	N = 250	Crude OR (95 % CI)	p value^#^	Adjusted OR^a^ (95 % CI)	p value^#^ adjusted
Female	87	2.7 (1.3–5.3)	0.05			86	1.3 (0.6–2.8)	0.5		
Age (continuous variable)	254	1.0 (1.0–1.0)	0.4			250	1.0 (1.0–1.1)	0.03	1.0 (1.0–1.0)	0.5
Time from diagnosis to examination (continuous variable)	254	1.1 (1.0–1.3)	0.2			250	1.0 (0.8–1.1)	0.7		
Unemployed	135	2.5 (1.3–4.9)	0.006	2.3 (1.1–4.8)	0.03	135	4.2 (2.1–8.4)	<0.001	4.3 (2.0–9.0)	<0.001
BMI > 30.0 kg/m^2^	76	3.1 (1.7–5.7)	<0.001	2.3 (1.1–4.2)	0.005	75	3.3 (1.8–6.1)	0.003	2.5 (1.3–5.0)	0.008
Current or previous daily smoking	131	1.2 (0.7–2.2)	0.5			127	1.2 (0.7–2.2)			
Still on anticoagulation	74	0.8 (0.4–1.5)	0.4			72	1.2 (0.6–2.3)	0.6		
No ECS after DVT	21	1.1 (0.3–3.4)	0.9			18	0.7 (0.2–2.4)	0.6		
COPD	13	1.6 (0.5–5.7)	0.4			12	1.7 (0.5–6.0)	0.4		
Heart failure	12	1.2 (0.3–4.8)	0.7			12	3.7 (1.1–11.9)	0.03		
Current or previous cancer	24	1.3 (0.5–3.4)	0.6			24	2.3 (0.9–5.4)	0.08		
Proximal DVT^b^	161	0.8 (0.4–1.6)	0.6			159	0.9 (0.5–1.8)	0.8		
PTS	121	13.9 (5.7–34.1)	<0.001	11.0 (4.6–29.7)	<0.001	120	5.9 (2.9–11.9)	<0.001	4.8 (2.3–10.0)	<0.001
Multiple DVTs	79	1.4 (0.8–2.7)	0.3			71	1.4 (0.7–2.6)	0.3		
Provoked DVT	119	0.8 (0.4–1.5)	0.5			116	1 (0.5–1.7)	0.9		

*DVT* deep vein thrombosis, *ECS* elastic compression stockings, *BMI* body mass index, *PTS* post thrombotic syndrome, *COPD* chronic pulmonary obstructive disease, *Impaired HRQoL* EQ-5D index value <25th percentile in patient group (0.72) and EQ VAS value <25th percentile in patient group (60)

^#^Wald test

^a^Adjusted for all other significant variables in the multivariate model

^b^In or above popliteal vein

In the multiple logistic regression models of EQ-5D index values and EQ VAS, PTS and obesity remained significantly associated with impaired HRQoL, while unemployment remained significant as measured by EQ VAS alone (Table 4).

The cut-off for impaired HRQoL was 41.7 and 40.9 for VEINES-QOL and VEINES Sym, respectively. In the simple analysis female gender, obesity, and PTS were found to be associated with impaired VEINES-QOL (Table 5), whereas only PTS and obesity were associated with impaired VEINES-Sym.

**Table 5:** Crude- and adjusted odds ratios (OR) for impaired HRQoL assessed by VEINES-QOL

Variable	VEINES-QOL				
	N = 195	Crude OR (95 % CI)	p value^#^	Adjusted OR^a^ (95 % CI)	p value^#^ adjusted
Female	67	2.0 (1.1–3.8)	0.025	2.5 (1.1–6.0)	0.04
Age (continuous variable)	195	1.0 (1.0–1.0)	1.0		
Time from DVT to examination (continuous variable)	195	1.0 (0.9-1.2)	0.8		
Unemployed	108	1.8 (1.0–3.4)	0.07		
BMI > 30.0 kg/m^2^	56	5.0 (2.6–9.7)	<0.001	4.1 (1.7–9.7)	<0.001
Current or previous daily smoking	103	1.0 (0.5–1.7)	0.8		
Still on anticoagulation	52	1.4 (0.7– 2.7)	0.3		
No ECS after DVT	14	0.5 (0.1–2)	0.4		
COPD	8	1.2 (0.3–5.4)	0.8		
Heart failure	10	9.2 (1.9–44.8)	0.06		
Current or previous cancer	13	0.9 (0.3–3.1)	0.9		
Proximal DVT^b^	120	0.8 (0.5–1.7)	0.7		
PTS	91	28.7 (11.3–73.0)	<0.001	28.2 (10.6–75.0)	<0.001
Multiple DVTs	59	1.8 (1.0–3.5)	0.06		
Provoked DVT	64	1.1 (0.6–2.1)	0.7		

*DVT* deep vein thrombosis, *ECS* elastic compression stockings, *BMI* body mass index, *PTS* post thrombotic syndrome, *COPD* chronic pulmonary obstructive disease, *Impaired HRQoL* VEINES-QOL <25th percentile in patient group (41.7)

^#^Wald test

^a^Adjusted for all other significant variables in the multivariate model

^b^In or above popliteal vein

In the multiple logistic regression analysis, only PTS and obesity were found as independent predictors of reduced QoL assessed by VEINES-QOL (Table 5) and VEINES Sym (not shown).

## Discussion

In this population-based cross-sectional study HRQoL was assessed by EQ-5D and VEINES-QOL/Sym. HRQoL assessed by EQ-5D was impaired among DVT patients compared to population norms and buddy controls. To our knowledge, no other studies have compared long-term HRQoL in an unselected DVT population to age- and sex-matched controls or population norms using EQ-5D.

The study showed that patients reported more problems in all the five items of EQ-5D compared to buddy-controls. Of these 5 items pain/discomfort was most common complaint in the patient group and was twice as prevalent as in the control group. This may be due to pain being a symptom of PTS (Kahn et al. 2000). The increased prevalence of patients indicating problems in the dimensions of mobility and usual activities may also be explained by restrictions due to symptoms of PTS. Anxiety was also more prevalent among patients. The latter is in contradiction to a study by van Korlaar et al. (van Korlaar et al. 2004) using SF-36, where no difference in mental component scores between DVT patients and population norms was found. Several factors may explain the reason for increased anxiety among patient, including fear of bleeding in those receiving anticoagulation or fear of recurrence in those who are not anticoagulated. These factors have been shown in other studies to influence HRQoL many years after a DVT (Casais et al. 2005).

In line with other reports our study confirms the negative impact of PTS on HRQoL (Kahn et al. 2000). PTS was associated with impaired HRQoL assessed by all questionnaires. PTS can influence HRQoL in several ways: it may limit the patients’ working ability, cause persistent pain, have cosmetic effect(s), and/or restrict patients’ activity of daily living (Kahn et al. 2000). Since treatment options for PTS once established are limited (Kahn et al.  2014), measures to prevent PTS are of importance in DVT patients. Strategies for prevention of PTS include ensure optimal anticoagulation, use of elastic compression stockings and catheter directed thrombolysis (Kahn et al.^2014^). However, the effect of compression stockings on the incidence of PTS is uncertain (Kahn et al. 2007) and catheter directed thrombolysis and other interventional treatment is not currently recommended for routine use for the purpose of PTS prevention in the general DVT patient population (Kahn et al.^2014^).

Parallel with other studies we found obesity to be associated with impaired HRQoL assessed by EQ-5D (Han et al. 2009). Obesity is known to affect physical activity and bodily pain and have a significant negative impact on the physical wellbeing (Han et al. 2009). We also found that obesity was associated with impaired HRQoL assessed by both VEINES subscales. In our study, however, obesity was an independent predictor of impaired HRQoL. It is conceivable that excess body weight might increase venous pressure and promote valvular reflux in already compromised veins, impairing HRQoL scores measured by VEINES QOL/Sym. The role of weight reduction in the management of obese DVT patients should be evaluated.

In line with previous studies, we found that unemployment was associated with poorer HRQoL assessed by EQ-5D (Garcia-Gordillo MA, Adsuar JC, Olivares PR. Normative values of EQ-5D-5L: in a Spanish representative population sample from Spanish Health Survey 2011). In the disease-specific VEINES-QOL/Sym employment status was not associated with impaired HRQoL. This seems logical as the disease-specific tool captures different aspects of HRQoL from the generic questionnaire. There was a trend towards female gender being a predictor of impaired HRQoL assessed by both EQ-5D index value and VEINES-QOL; however, it failed to remain significant when controlling for other factors (Tables 4, 5).

The association between recurrent DVT and HRQoL reported in the literature is inconsistent (Prandoni et al. 1996; Kahn et al. 2008). In this study we did not find any association between recurrent DVT and reduced HRQoL. In most previous reports, proximal DVT was found as a predictor of impaired disease specific HRQoL (Kahn et al. 2005, 2008; Rabinovich and Kahn 2014). However, in line with Roberts et al. (2014) we did not find an association between proximal extension of DVT and impaired HRQoL. In contrast with other studies (Kahn et al. 2004, 2008) we found that the presence of comorbidities (heart failure and COPD) were not associated with impaired HRQoL, when adjusting for other factors, probably because very few patients had these comorbidities in our study sample leading to low power.

Interpretation of clinically relevant differences in scores is an important issue in HRQoL measurements. Nonetheless, there is no definitive consensus on the most appropriate method for assessing meaningful differences. In this study, we followed criteria established and compared to other measures of health (SF-36) in previous investigations of MIDs for EQ-5D-3L (Walters and Brazier 2005; Pickard et al. 2007), although other methods have been reported and used in other studies (Jansson et al. 2009).

The major strength of our study is recruitment of unselected DVT patients and the inclusion of buddy controls. Although many patients lacked a buddy control, the age and sex distribution of patients with buddy controls and those without were the same. Comparison with the control group therefore seems to reflect a real difference in HRQoL between patients with and with out a history of DVT. The main limitations of this study are that patients were identified retrospectively and only 61 % surviving patients participated in the study. We therefore cannot rule out the possibility of recruiting more patients with PTS or other complaints. However, since the prevalence and distribution of PTS, comorbidities and localization of DVT was comparable to that reported in other studies, we consider the risk of such bias as low (Kahn et al.  2014; Kahn 2006).

## Conclusions

We conclude that DVT patients participating in our study had impaired long-term HRQoL as compared with buddy controls and population norms. PTS and obesity were the most important predictors for impaired HRQoL as measured by both generic- and disease-specific tools. PTS and obesity are modifiable factors; our findings thus emphasize the importance of measures to prevent PTS and obesity.

## Abbreviations

COPD: chronic obstructive pulmonary disease
CVDL: chronic venous diseases of the leg
DVT: deep vein thrombosis
ECS: elastic compression stockings
EQ-VAS: EQ Visual Analogue Scale
EQ-5D: descriptive system of health-related quality of life states consisting of five dimensions
HRQoL: health related quality of life
IQR: interquartile range
MID: minimal clinical important difference
PROMs: patient reported outcome forms
PTS: post thrombotic syndrome
Obesity: body mass index >30/m^2^
SD: standard deviation
TTO: time-trade-off
VEINES-QOL/Sym: disease-specific quality of life instrument for use in venous diseases of the leg
